# Integrative microRNA and mRNA deep-sequencing expression profiling in endemic Burkitt lymphoma

**DOI:** 10.1186/s12885-017-3711-9

**Published:** 2017-11-13

**Authors:** Cliff I. Oduor, Yasin Kaymaz, Kiprotich Chelimo, Juliana A. Otieno, John Michael Ong’echa, Ann M. Moormann, Jeffrey A. Bailey

**Affiliations:** 1 Center for Global Health Research, Kenya Medical Research Institute, Kisumu, Kenya; 2 Department of Biomedical Sciences and Technology, Maseno University, Maseno, Kenya; 3 Department of Bioinformatics & Integrative Biology, University of Massachusetts Medical School, Worcester, MA USA; 4 Jaramogi Oginga Odinga Teaching and Referral Hospital, Ministry of Health, Kisumu, Kenya; 5 Department of Molecular Medicine, University of Massachusetts Medical School, Worcester, MA USA; 6 Division of Transfusion Medicine, Department of Medicine, University of Massachusetts Medical School, 368 Plantation St. Albert Sherman Building 41077, Worcester, MA 01605 USA

**Keywords:** Endemic Burkitt lymphoma, miRNA, mRNA, RNA sequencing, Lymphomagenesis

## Abstract

**Background:**

Burkitt lymphoma (BL) is characterized by overexpression of the *c-myc* oncogene, which in the vast majority of cases is a consequence of an IGH/MYC translocation. While *myc* is the seminal event, BL is a complex amalgam of genetic and epigenetic changes causing dysregulation of both coding and non-coding transcripts. Emerging evidence suggest that abnormal modulation of mRNA transcription via miRNAs might be a significant factor in lymphomagenesis. However, the alterations in these miRNAs and their correlations to their putative mRNA targets have not been extensively studied relative to normal germinal center (GC) B cells.

**Methods:**

Using more sensitive and specific transcriptome deep sequencing, we compared previously published small miRNA and long mRNA of a set of GC B cells and eBL tumors. MiRWalk2.0 was used to identify the validated target genes for the deregulated miRNAs, which would be important for understanding the regulatory networks associated with eBL development.

**Results:**

We found 211 differentially expressed (DE) genes (79 upregulated and 132 downregulated) and 49 DE miRNAs (22 up-regulated and 27 down-regulated). Gene Set enrichment analysis identified the enrichment of a set of MYC regulated genes. Network propagation-based method and correlated miRNA-mRNA expression analysis identified dysregulated miRNAs, including miR-17~95 cluster members and their target genes, which have diverse oncogenic properties to be critical to eBL lymphomagenesis. Central to all these findings, we observed the downregulation of *ATM* and *NLK* genes, which represent important regulators in response to DNA damage in eBL tumor cells. These tumor suppressors were targeted by multiple upregulated miRNAs (miR-19b-3p, miR-26a-5p, miR-30b-5p, miR-92a-5p and miR-27b-3p) which could account for their aberrant expression in eBL.

**Conclusion:**

Combined loss of p53 induction and function due to miRNA-mediated regulation of *ATM* and *NLK*, together with the upregulation of *TFAP4*, may be a central role for human miRNAs in eBL oncogenesis. This facilitates survival of eBL tumor cells with the IGH/MYC chromosomal translocation and promotes MYC-induced cell cycle progression, initiating eBL lymphomagenesis. This characterization of miRNA-mRNA interactions in eBL relative to GC B cells provides new insights on miRNA-mediated transcript regulation in eBL, which are potentially useful for new improved therapeutic strategies.

**Electronic supplementary material:**

The online version of this article (10.1186/s12885-017-3711-9) contains supplementary material, which is available to authorized users.

## Background

Endemic Burkitt lymphoma (eBL) is a germinal center (GC) B-cell cancer occurring at a high incidence in sub-Saharan Africa. This pediatric cancer was first described by Denis Burkitt in association with rainfall and later linked with increased *P. falciparum* malaria prevalence [1, 2]. What became recognized as an ubiquitous childhood virus, Epstein-Barr Virus (EBV) was also first described within an eBL tumor, and thus became the first virus associated with a human malignancy [3, 4]. While generally sensitive to cytotoxic chemotherapies, some tumors remain or become refractory, which contributes to poor outcomes for these children [5, 6]. It is therefore critical to elucidate all mechanisms involved in eBL pathogenesis in order to identify molecular targets for both early detection, prognostic indicators, and more effective therapy to improve outcomes for these children.

BL is subdivided into an EBV-associated endemic form (eBL) in Africa (also in New Guinea), a sporadic form (sBL) that is most prevalent in developed countries, and an HIV-associated or immunodeficiency-related BL form (id-BL). All forms of BL are characterized by overexpression of the *MYC* gene, a transcription factor and proto-oncogene, that has roles in cell cycle progression, apoptosis and central to B cell transformation [7]. This overexpression is most often a consequence of a translocation involving chromosomes 8 and 14 approximating the *IGH* enhancer to an intact *MYC* locus [8, 9]. Less common translocations can involve either of the light chain enhancers positioned next to *MYC* or the direct mutation of the gene leading to its overexpression [10–12]. Simple overexpression of *MYC* is not in and off itself transformative in normal cells as multiple mechanisms and checkpoints exist that counteract aberrant *MYC* expressions triggering apoptosis [13, 14]. This suggests that there are likely additional genetic and epigenetic changes to fully potentiate the oncogenic transformation. This multi-factorial concept has been strongly supported by a number of studies demonstrating further driver mutations and epigenetic changes [15–19], that play important roles in tumor proliferation, maintenance and abrogating checkpoints in the face of *MYC* overexpression [16, 17]. However, the exact pattern and combinations of driver mutations and epigenetic changes necessary or sufficient for lymphomagenesis has not been fully elucidated.

Endemic BL, like all other forms of BL, is thought to originate from GC B cells based on the expression of V-region genes diversified by somatic mutations in conjunction with its extra-nodal presentation [20]. A GC program is supported by the detection of somatic mutations in the rearranged V region genes that are characteristic of GC B-cell differentiation [20, 21]. While it is unclear if BL cells truly traverse the GC, it is clear that GC B cells are their best normal counterpart and that BL is likely an oncogenically altered GC program [22] in which GC-restricted transcription factors have powerful oncogenic influence. The expression of protein coding genes and polyadenylated transcripts have provided initial key insights into tumor dysregulation. However, transcriptome expression differences that would facilitate oncogenesis have not been fully explored in eBL. MicroRNAs, being one of the key transcriptome components that have not been carefully examined in primary tumors, may contribute significantly to altered gene expression and initiate lymphomagenesis.

MicroRNAs (miRNAs) are a recently discovered class of small noncoding RNAs with 18 to 24 nucleotides, that regulate gene expression post-transcriptionally by binding to mRNAs with complementarity [23, 24]. They have been described as managers of gene expression by targeting mRNAs for degradation or translational repression and play a fundamental role in many cellular processes including proliferation, apoptosis, and cell survival that are often key in oncogenesis [25]. Dysregulation of miRNAs have been found to initiate malignant phenotypes, resulting in development of various cancers [26, 27]. MiRNA expression profiling studies can be especially rich in biological information, as variations in expression of hundreds of protein-coding genes may be captured in the expression patterns of one or a few miRNAs that regulate them [26, 27]. To date, the global miRNA and mRNA expression patterns of eBL have not been interrogated. An evaluation of aberrant miRNA and mRNA expression changes in eBL, compared to its normal counterpart, could provide an insight into mechanisms involved in eBL genesis and progression. The identification of oncomirs and tumor suppressor miRs, would be of potential value in the development of novel therapeutic agents targeting miRNAs via mimics or antagomirs.

Although there are studies on mRNA/miRNA profiling, and reports on BL and other non-Hodgkins lymphomas [28–34], a combined analysis of mRNA and miRNA expression patterns of eBL has not been performed using more sensitive and specific next-generation deep sequencing. An integrative analysis of differentially regulated miRNA and mRNA expression in eBL tumors compared to GC B cells will help us better understand the mechanisms involved in oncogenesis and identify key miRNAs and miRNA-mRNA interactions that may underlie eBL lymphomagenesis.

## Methods

### Sample collection and ethical approval

We collected Fine Needle Aspirates (FNA) of the primary tumors from children aged between 5 and 12 years diagnosed with endemic BL. The biopsy samples were prospectively collected between 2009 and 2012 prior to chemotherapy treatment at Jaramogi Oginga Odinga Teaching and Referral Hospital (JOOTRH) located in Kisumu City, a regional referral hospital for pediatric cancer cases in western Kenya. Touch prep slides were made from the FNA biopsies and stained using May-Grünwald Giemsa (MGG) staining for morphologic diagnosis. A portion of the biopsy was transferred into RNAlater equilibrated for a day at 4 °C and stored long-term at −20 °C.

Ethical review and approval for this study was obtained from the Institutional Review Board at the University of Massachusetts Medical School, USA and the Scientific and Ethics Review Unit (SERU) at the Kenya Medical Research Institute (KEMRI), Kenya. Parents and legal guardians of the study participants provided written informed consent.

In order to compare eBL to their presumed normal counterpart, GC B cells, we reanalyzed published publicly available miRNAseq and mRNAseq GC B-cell datasets from three previous publications [35–37] and databases (https://ega-archive.org/datasets/EGAD00001002452). The raw miRNAseq and mRNAseq fastq read files of the sorted GC B-cells samples were downloaded through the Gene Expression Omnibus (GEO) archive through accession GSE22898 and the Blueprint consortium, dataset ID: EGAD00001002452.

### RNA and small RNA isolation

Total RNA and Small RNA molecules were extracted from eBL FNA samples in RNAlater using the AllPrep DNA/RNA/miRNA Universal kit (Qiagen) according to manufacturer’s instructions. Small RNA abundance and integrity were determined after isolation using Nanodrop-ND-1000 spectrophotometer (Thermo Fisher Scientific, Waltham, Massachusetts, USA) and an Agilent 2100 Bioanalyzer (Agilent Technologies, Santa Clara, CA), respectively. Only samples with a miRNA concentration > 10 ng/μl and total RNA RIN (RNA integrity number) > 8.0 were considered for small RNA library preparations and sequencing, as a result, of the 28 samples only 17 were considered for miRNA library preparation. All isolated nucleic acids were stored at −80 °C.

### MicroRNA sequencing

Seventeen indexed miRNA libraries were prepared using the Illumina Truseq Small RNA Library Preparation Kit (Illumina Inc., San Diego, CA, USA) following the manufacturer’s protocol. The purified small RNA libraries were quantified using the Agilent High Sensitivity DNA Kit (Agilent Technologies, Colorado Springs, CO, USA) and their size distribution was also confirmed. The miRNA libraries were pooled in equimolar concentrations and sequenced on one lane of an Illumina HiSeq 2000 platform (Illumina Inc., San Diego, CA, USA). The fastq files were produced using the CASAVA pipeline v2.0 (Illumina Inc., San Diego, CA, USA) and all generated sequence data can be accessed in NCBI dbGAP accession number: phs001282.v2 [18].

Preliminary quality control analysis of the 17 fastq files from the eBL patients and the 4 fastq files from GC B cells obtained from Gene Expression Omnibus (GEO) archive, were carried out with FASTQC software v0.10.0 [38]. Cutadapt v1.1 [39] was then used to trim off the 3′-adaptor sequences from the sequencing reads. Novobarcode [40] was then used to de-multiplex the 17 eBL samples based on the 6-nucleotide barcode that was added to the smallRNA sequencing library of each sample. Reads shorter than 18 nucleotides after adaptor trimming and barcode removal were discarded. Reads passing all the above filters were aligned to human genome (hg19) using bowtie [41]. The resulting sequences were subjected to our computational pipeline, which consists of a number of in-house made scripts using miRDeep2 [42] to determine the miRNA counts for each of the samples.

### RNA sequencing

Briefly, starting with 1-5 μg total RNA, we prepared strand-specific RNAseq libraries following the protocol from Zhang et al. [43] combined with mRNA enrichment with oligo-dT using Dynabeads mRNA purification kit (Life Technologies). Final library qualities were confirmed with Bioanalyzer High sensitivity DNA kit (Agilent) and sequenced with paired end read (2x100bp) using multiple lanes of Illumina HiSeq 2000 (Illumina Inc., San Diego, CA, USA). Data can be accessed at dbGAP with accession number phs001282.v1.

### Differential gene expression analysis

After quality assessment and preprocessing the raw sequencing reads, we aligned mRNA read pairs to a transcriptome index built by RSEM [44] using Gencode v19 protein coding transcript annotations and hg19 genomic sequence. To perform differential gene expression test between 28 eBL tumors and 5 GC B-cells, we used edgeR [45] in R computing environment. To be able to account for the batch variables and unknown factors while testing for the differential expression between the eBL tumors and GC B-cell RNA expression data from another dataset, we estimated the number of latent factors for every comparison separately using svaseq [46] while preserving the variation of interest (Additional file 1). We then incorporated these surrogate variables into the testing model for edgeR. *P*-values were adjusted for multiple testing with the Benjamini and Hochberg (1995) approach for adjusting the false discovery rate (FDR) and adjusted *p*-values were filtered at 0.01. Significantly differentially expressed (DE) mRNAs had Benjamini-Hochberg (BH) multiple test corrected *P*-values < 0.01.

### Gene set enrichment analysis

We performed a standard gene set enrichment analysis (GSEA) using the GSEA module implemented by Broad Institute, Cambridge, MA. GSEA was performed on normalized expression data and on data after surrogate variable analysis as described by Kaymaz Y. et al [18]. For a ranking metric, we used signal to noise value of each gene, and performed a permutation test for FDR by permuting sample phenotypes (eBL tumor cells and GC B cells). The analysis included standard gene sets of hallmark and oncogenic signatures as well as the curated C2 gene sets from the Molecular Signatures Database (v5.0 MSigDB).

### Differential miRNA expression analysis

Differential miRNA expression was performed between the 17 eBL tumor cells and 4 GC B cells. This expression analysis of miRNA-Seq data was also performed using the R/Bioconductor package edgeR [45]. First, we counted the number of reads uniquely mapped to miRNA regions according to the reference database miRBase [47]. Only miRNAs that had at least 10 counts per million in at least half of the samples were analyzed for evidence of differential gene expression. The biological reason for this is that a miRNA must be expressed at some minimal level before it is likely to affect gene regulation. The statistical reason was that very low counts would provide little statistical information to distinguish between the null and the alternative hypothesis [47]. We also applied svaseq [46] to account for the batch variables and unknown factors while preserving the variation of interest for the differential expression analysis. We then incorporated these surrogate variables into the testing model for edgeR. P-values were also adjusted for multiple testing with the Benjamini and Hochberg (1995) approach for adjusting the FDR and adjusted *p*-values were filtered at 0.01. Significantly DE miRNA also had BH multiple test corrected *P*-values <0.01.

### Network propagation method to infer the perturbed miRNA regulatory network using differential gene expression data

The network propagation based method (NP-method) [48], was used to infer the key miRNA regulatory networks whose perturbation is most likely to induce the observed gene expression changes in eBL compared to their normal counterpart. By integrating eBL differential gene expression data with prior biological knowledge of miRNA-target interactions [49] and the TF (Transcription factor)-gene regulatory network (HTRIdb) [50], a network-based random walk with restart (RWR) plus forward searching algorithm [51] was carried out to calculate the network perturbation effect score (NPES) of miRNAs and extract their leading-edge target genes. To avoid bias towards miRNAs with a large target set, gene set permutation based analysis repeated 1000 times was performed to normalize the score and estimate the *p*-value for each miRNA.

### MicroRNA target identification

miRNAs regulate expression of specific genes via hybridization to mRNA transcripts to promote RNA degradation, inhibit translation or both [52]. Identification of target genes of the aberrantly expressed miRNAs is important for understanding the regulatory networks associated with eBL development. To investigate the biological relevance of the identified DE miRNAs, we identified all the validated target genes for the DE miRNAs using the validated target module of the miRWalk2.0 [53, 54] database.

### MicroRNA-mRNA pairs of interest

To identify miRNA-mRNA pairs of interest, we first identified the DE validated target genes of the DE miRNAs, that exhibited an inverse expression change to the miRNA (Pearson correlation, *P* = 0.05). We tested if the miRNA-mRNA pairs are of potential biological significance or by chance. To achieve this, we performed a permutation test of significance repeated 10,000 times. The permutation tested whether the number of miRNA-mRNA pairs were more than would be expected by chance.

### GO and KEGG pathway enrichment analysis

For functional analyses of the miRNA targets, Gene ontology (GO) term analysis was applied to organize genes into categories based on biological processes, cellular components and molecular functions. Biological pathways defined by Kyoto Encyclopedia of Genes and Genomes (KEGG) analysis were identified by DAVID (Database for Annotation, Visualization and Integrated Discovery) software [55]. DAVID online was used to provide a set of functional annotations of a large number of genes. *P*-values of each pathway were adjusted using the Benjamini-Hochberg method to control the FDR. In the current study, GO terms and signaling pathways were selected with the threshold of significance being defined as *P* < 0.01 and FDR < 0.05.

## Results

### Expression of germinal center (GC) B cell differentiation genes in eBL tumors

Using publically available miRNA and mRNA data from our published eBL [18, 56] and published normal GC B cells [35–37], we first examined the mRNA gene expression to ensure proper signatures consistent with the described tumor and GC B cell expression phenotype. The expression levels of B cell differentiation genes in eBL tumor cells were at comparable levels to the GC derived B cells. RNA expression counts of key GC transcription factors (BCL6 and PAX5) were well expressed while plasma cell genes (BLIMP1 and IRF4) were at low levels. Overall, this supports a GC B-cell like tumor phenotype and the validity of further comparisons between these malignant and normal GC B cells (Additional file 2).

### Gene expression profiling comparing germinal center B cells and endemic BL

**Fig. 1:**
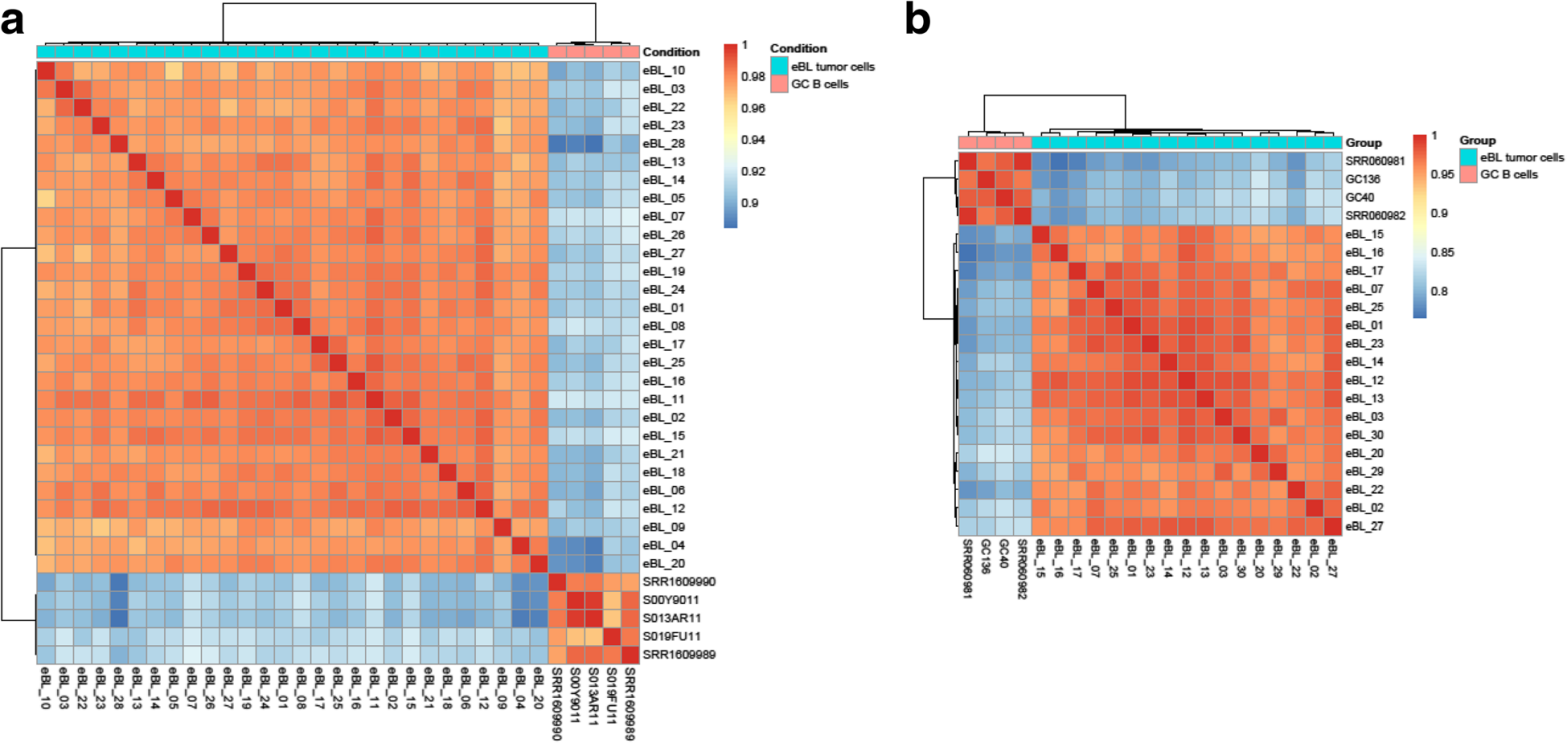
Sample to sample hierarchal clustering of eBL tumor cells and GC B cells based on **a** mRNA expression profiles, **b** miRNA expression profiles with highest correlation of variation (CV) values (calculated using regularized log transformed mRNA and miRNA expression values)

**Fig. 2:**
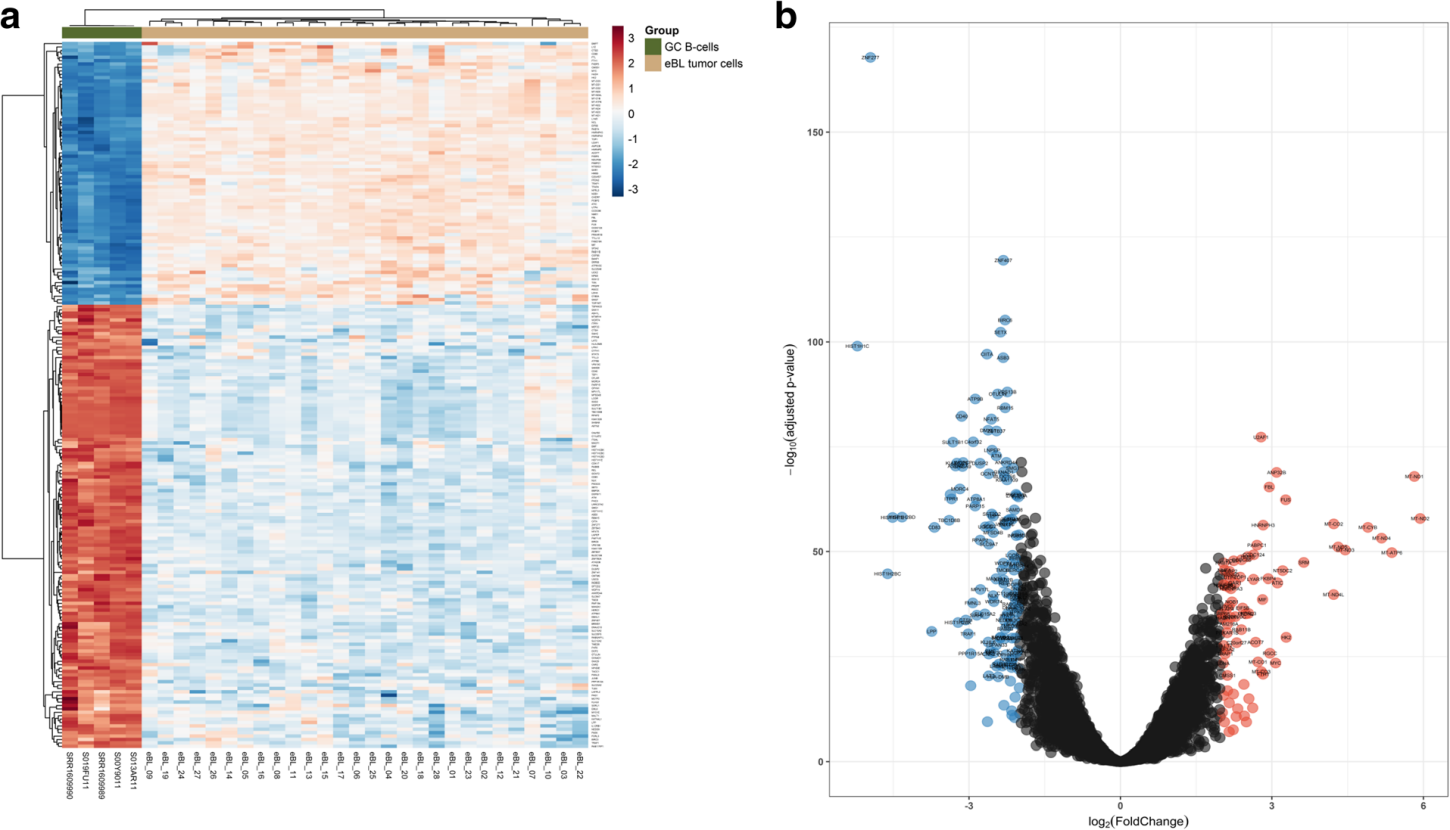
Differentially expressed mRNAs in eBL compared to GC B cells. **a** Heatmap of differentially expressed (DE) miRNAs between eBL tumor cells and GC (Germinal center) B cells. The heatmap shows the hierarchical clustering based on the expression profiles of the 211 DE genes with at least 2-fold difference in expression compared to their normal counterpart. **b** Volcano plot representing the significance genes (−log of the adjusted *p*-value) vs the fold change difference in eBL compared to GC B-cells. The red and blue colored circles represent genes which are DE with *p* < 0.01 and FDR < 0.01. The 132 down-regulated genes in eBL are colored blue (have a negative fold-change value) while the 79 up-regulated genes in eBL are colored red (positive fold-change value)

To identify genes that may contribute to the oncogenic phenotype of eBL, gene expression profiling was conducted on 28 eBL of our newly sequenced tumor samples [18] and 5 previously published GC B cells [35–37]. As shown in Fig. 1, hierarchical clustering of most variant genes revealed a clear separation of the two groups where the eBL samples are clearly differentiated from their normal counterparts. Performing differential expression analysis between eBL and normal GC B cells, we identified 211 differentially expressed (DE) genes using stringent thresholds (logFC > 2, *p*-value < 0.01 and FDR < 0.01) (Fig. 2, Additional file 3). Of these, 132 genes were downregulated and 79 were upregulated in eBL compared to their normal GC counterparts (Additional file 3). Among the upregulated genes was *MYC*, whose over-expression is central to BL oncogenesis (logFC = 3.07, *p*-value = 5.50E-24 and a FDR = 1.15E-22). Also among the upregulated genes were mitochondrial protein coding genes (*MT-ND3, MT-ND4L, MT-ND4, MT-ND2, MT-CYB, MT-ND1, MT-CO1* etc), which may be as a result of the elevated metabolism characteristic of cancer cells to sustain their survival. We observed the upregulation of *TFAP4* (Transcription factor activating enhancer-4)/*AP4*, a direct transcriptional target of *MYC* to induce cell cycle progression, (logFC = 2.12, *p*-value = 1.18E-35 and FDR = 6.35E-34). We also observed the downregulation of the protein kinases *ATM* (ataxia-telangiectasia mutated) (logFC = −2.46, *p*-value < 0.0001 and FDR < 0.00001), an activator of the DNA damage response in the face of DNA double strand breaks (DSBs), and *NLK* (nemo-like kinase) (logFC = −2.55, *p*-value < 0.0001 and FDR < 0.0001), a p53 activator, in eBL tumor cells.

**Fig. 3:**
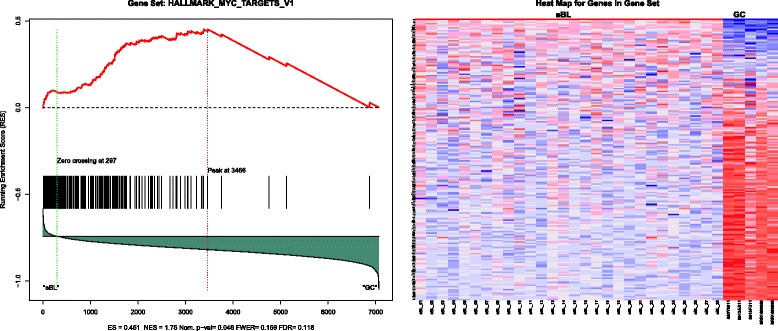
Gene set enrichment plot and expression heatmap of corresponding genes in the enriched gene set. Left panels include the running enrichment score throughout the gene set and projection of genes in the geneset to the complete list of genes rank ordered based on signal to noise ratio. On the expression heatmap (columns are eBL tumors and GC B cells, rows are genes in the gene set), dark red represent higher expression while dark blue lower expression. Genes in this enrichment are a set of genes regulated by MYC in eBLs tumor cells relative to GC B cells (ES = 0.45, Nominal *P* = 0.046, FDR q = 0.118)

To identify more subtle changes in overall pathways and functional sets of genes, we also performed gene set enrichment analysis (GSEA). The analysis, using a FDR < 0.25, detected only one enriched gene set. This was the gene set HALLMARK_MYC TARGETS_V1 (a set of genes regulated by *MYC*) (Fig. 3; Additional file 4), which again highlights MYC’s pivotal role in eBL oncogenesis. This comparison confirms that our eBL dataset is consistent with expected differences between normal and cancerous B cells.

### MiRNA expression profiling in endemic BL

**Fig. 4:**
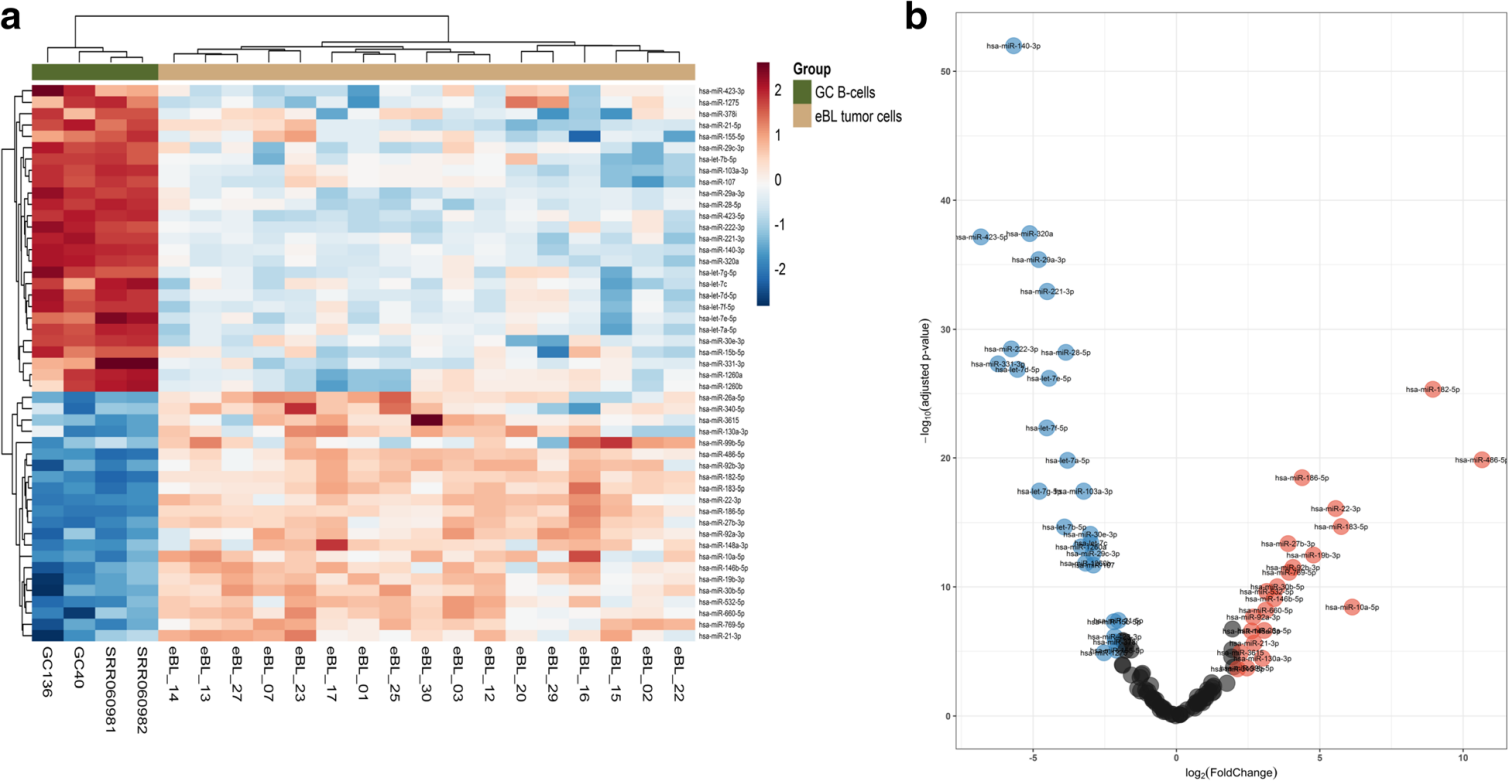
Differentially expressed miRNAs in eBL compared to GC B cells. **a**. Heatmap of differentially expressed (DE) miRNAs between eBL tumor cells and GC (Germinal center) B cells. The heatmap shows the hierarchical clustering based on the expression profiles of the 49 DE miRNAs with at least 2-fold difference in expression compared to their normal counterpart. **b**. Volcano plot representing the significance miRNAs (−log of the adjusted p-value) vs the fold change difference in eBL compared to GC B-cells. The red and blue colored circles represent miRNAs which are DE with *p* < 0.01 and FDR < 0.01. The 27 down-regulated miRNAs in eBL are colored blue (have a negative fold-change value) while the 22 up-regulated miRNAs in eBL are colored red (positive fold-change value)

Next, to identify differences in miRNA-mRNA regulatory networks, we profiled the miRNA expression of 17 eBL tumor samples compared to 4 GC B cells. Hierarchical cluster analysis on the expression profile of the most variant miRNAs separated the GC B cells from the eBL tumor cells (Fig. 1). We identified 49 miRNAs to be significantly DE (logFC > 2, *p*-value < 0.01 and FDR < 0.01) between eBL and GC B cells. Of these, 27 miRNAs were downregulated and 22 were upregulated in eBL samples compared to GC B cells (Fig. 4, Table 1). For these 49 DE miRNAs, we used miRWalk2.0 to identify their validated mRNA targets (Additional file 5). Gene ontogeny and pathway enrichment analysis of the validated targets, revealed the enrichment of Pathways in Cancer (*p*-value < 0.01 and FDR < 0.01) as the top enriched KEGG pathway, and other cancer associated KEGG pathways (such as hsa05205:Proteoglycans in cancer, hsa05203:Viral carcinogenesis, hsa05220:Chronic myeloid leukemia) (Additional file 6). Of the DE miRNAs, there was a marked number targeting critical tumor suppressors (*PTEN*, *AXIN1*, *ATM*, *NLK*) and critical proto-oncogenes and tumor promoting genes such as *MYC* [57] (Additional file 5). For instance, the downregulated miRNAs included let-7 family members (let-7a-5p, let-7b-5p, let-7c, let-7d-5p, let-7e-5p, let-7f-5p, let-7 g-5p) (logFC < −2.5), which all target *MYC* gene for post-transcriptional regulation [58–62]. Among the upregulated miRNAs in eBL were members of the miR-17~92 cluster (miR-19b-3p, and miR-92a-3p) (logFC > 3), which target tumor suppressor genes such as *TP53* [63] and *ATM* (ataxia telangiectasia mutated) kinase [59, 64], respectively.

**Table 1:** Differentially expressed miRNAs between eBL tumor cells and GC B cells (logFC > 2, *p*-value < 0.01 and FDR < 0.01)

miRNA name	Log FC eBL versus GC B cells	BH adjusted *p*-value	FDR	Regulation eBL versus GC B cells
Upregulated miRs				
hsa-miR-486-5p	10.6609	1.38E-20	1.19E-19	Up
hsa-miR-182-5p	8.9478	4.55E-26	4.63E-25	Up
hsa-miR-10a-5p	6.1349	3.60E-09	1.19E-08	Up
hsa-miR-183-5p	5.7463	2.02E-15	1.19E-14	Up
hsa-miR-22-3p	5.5587	7.96E-17	4.95E-16	Up
hsa-miR-19b-3p	4.7807	3.32E-13	1.43E-12	Up
hsa-miR-186-5p	4.3815	3.43E-19	2.56E-18	Up
hsa-miR-92b-3p	4.0673	3.01E-12	1.16E-11	Up
hsa-miR-769-5p	3.9268	7.24E-12	2.70E-11	Up
hsa-miR-27b-3p	3.8964	4.25E-14	2.07E-13	Up
hsa-miR-30b-5p	3.5192	9.55E-11	3.45E-10	Up
hsa-miR-146b-5p	3.4102	8.07E-10	2.74E-09	Up
hsa-miR-532-5p	3.1493	2.26E-10	7.93E-10	Up
hsa-miR-660-5p	3.1183	6.35E-09	2.03E-08	Up
hsa-miR-26a-5p	3.0639	2.38E-07	6.65E-07	Up
hsa-miR-130a-3p	3.0018	3.50E-05	7.13E-05	Up
hsa-miR-21-3p	2.7137	2.29E-06	5.58E-06	Up
hsa-miR-92a-3p	2.6837	2.11E-08	6.58E-08	Up
hsa-miR-148a-3p	2.6221	2.61E-07	7.14E-07	Up
hsa-miR-99b-5p	2.4566	1.97E-04	3.74E-04	Up
hsa-miR-3615	2.2328	1.23E-05	2.65E-05	Up
hsa-miR-340-5p	2.1321	2.30E-04	4.30E-04	Up
Downregulated miRs				
hsa-miR-423-5p	−6.82082	6.92E-38	2.59E-36	Down
hsa-miR-331-3p	−6.22424	4.78E-28	6.70E-27	Down
hsa-miR-222-3p	−5.76142	3.31E-29	6.18E-28	Down
hsa-miR-140-3p	−5.67778	1.03E-52	1.16E-50	Down
hsa-let-7d-5p	−5.54828	1.29E-27	1.61E-26	Down
hsa-miR-320a	−5.12011	3.82E-38	2.14E-36	Down
hsa-miR-29a-3p	−4.80203	4.01E-36	1.12E-34	Down
hsa-let-7 g-5p	−4.78494	3.71E-18	2.46E-17	Down
hsa-let-7f-5p	−4.52246	4.60E-23	4.29E-22	Down
hsa-miR-221-3p	−4.50804	1.18E-33	2.64E-32	Down
hsa-let-7e-5p	−4.44258	6.34E-27	7.10E-26	Down
hsa-let-7b-5p	−3.90908	2.16E-15	1.21E-14	Down
hsa-miR-28-5p	−3.85689	6.16E-29	9.86E-28	Down
hsa-let-7a-5p	−3.79827	1.52E-20	1.22E-19	Down
hsa-miR-1260a	−3.32632	7.61E-14	3.55E-13	Down
hsa-miR-103a-3p	−3.23483	3.74E-18	2.46E-17	Down
hsa-miR-1260b	−3.16842	1.42E-12	5.91E-12	Down
hsa-miR-30e-3p	−3.00521	8.00E-15	4.27E-14	Down
hsa-let-7c	−2.98489	3.79E-14	1.93E-13	Down
hsa-miR-29c-3p	−2.90631	2.48E-13	1.11E-12	Down
hsa-miR-107	−2.89683	2.00E-12	8.00E-12	Down
hsa-miR-1275	−2.52971	1.30E-05	2.74E-05	Down
hsa-miR-15b-5p	−2.17524	4.93E-08	1.45E-07	Down
hsa-miR-423-3p	−2.15549	7.20E-07	1.92E-06	Down
hsa-miR-378i	−2.13256	1.86E-06	4.64E-06	Down
hsa-miR-155-5p	−2.07727	8.29E-06	1.82E-05	Down
hsa-miR-21-5p	−2.02446	4.01E-08	1.22E-07	Down

*Abbreviations*: *eBL* endemic Burkitt lymphoma, *GC* germinal center, *BH* Benjamini & Hochberg, *FC* Fold Change, *FDR* False discovery rate

### Prediction of miRNAs influencing aberrant gene expression in eBL using network propagation-based method

A miRNA could regulate gene expression in eBL cells either by directly targeting genes dysregulated in eBL or by targeting regulatory elements (such as transcription factors), whose impact may propagate across the whole regulatory network to influence eBL development. Thus, we used the mRNA expression data in a network propagation model [48] to identify miRNAs, whose expression change may contribute to the observed gene expression alterations in eBL tumor cells compared to their normal counterparts. MiRNA-target regulation information [49] and the transcription regulatory database (HTRIdb) was used to model the network effects of the miRNA dysregulation in eBL. The correlation between network effect of the miRNA perturbation and gene ranking was evaluated. This identified 12 eBL-related miRNA families significantly enriched (network perturbation effect score (NPES) >2, adjusted *p*-value < 0.05 and FDR < 0.1) in regulation of the aberrant gene expression profile in eBL (Additional file 7). The top ranked miRNAs (NPES > 2.8, *p* = 0.001 and FDR < 0.02) included, miR-19b-3p (miR-19ab family) and miR-92a/b-3p (miR-25/32/92abc/363/363-3p/367 family), were significantly upregulated in eBL tumor cells, and targets tumor suppressor genes such as *ATM* and *NLK*, which are observed to be downregulated in eBL. Overall, the enriched miRNAs (Additional file 7) are more likely to cause the observed differential gene expression in eBL, to supplement the aberrant molecular mechanisms involved in lymphoma development.

### Integration of miRNA and mRNA expression data

**Fig. 5:**
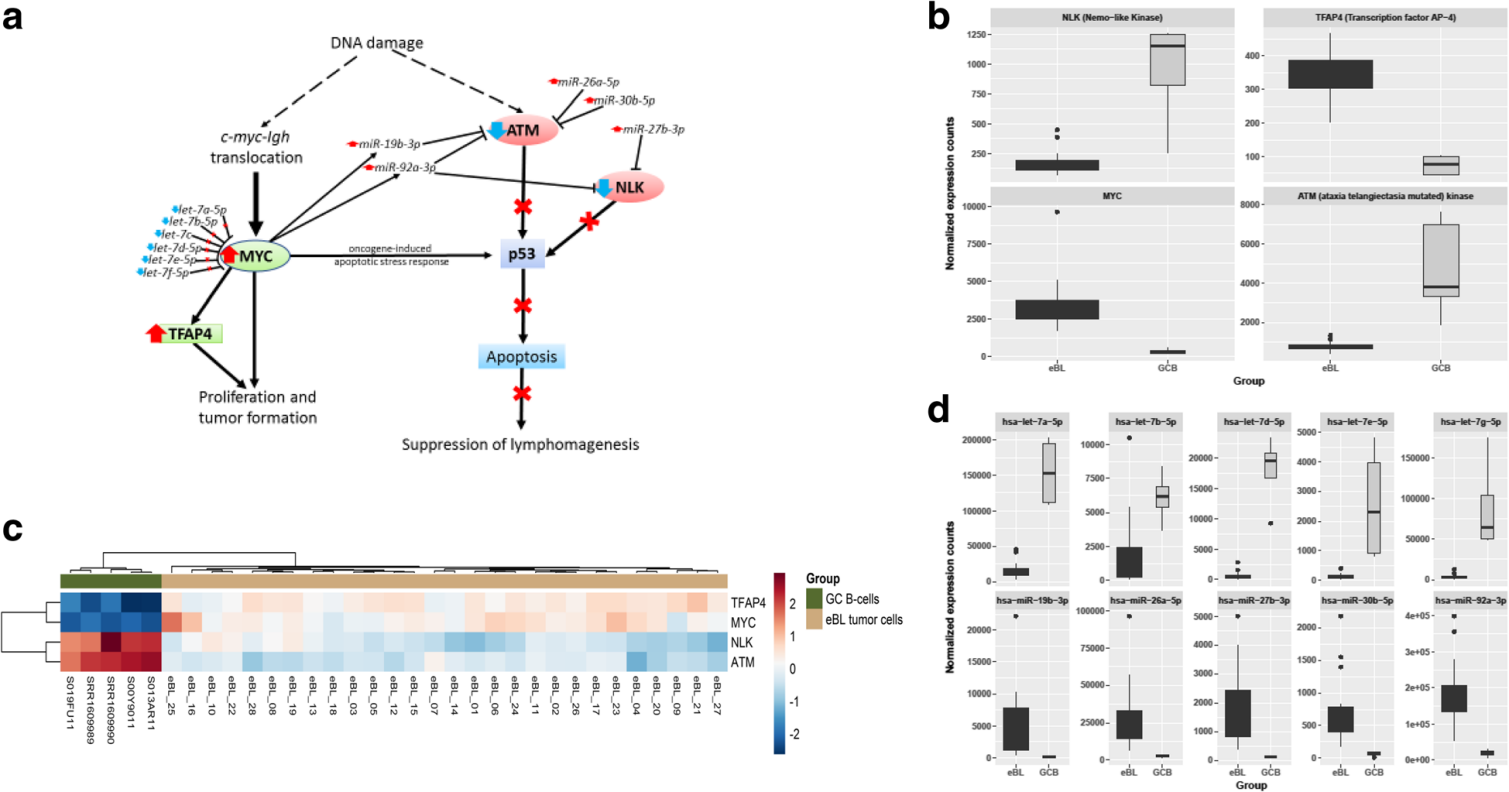
Aberrant transcriptome expression pivotal to eBL lymphomagenesis. **a** Schematic illustration of the aberrant gene expression and miRNA mediated regulatory changes that would initiate lymphomagenesis as a result of DNA damage. Combined loss of p53 function due to small interfering RNA-mediated regulation of *ATM* and *NLK* together with upregulation of TFAP4, would facilitate survival of cells with the *c-myc-Igh* chromosomal translocation and MYC induced cell cycle progression initiating eBL tumor development. ATM checkpoint kinase, transduces genomic stress signals to halt cell cycle progression in response to DNA damage. It is critical in the regulation of apoptosis and lymphomagenesis in *c-myc* induced lymphomas. *ATM* is downregulated in eBL and it is targeted by 4 miRs that are Upregulated in eBL. NLK is required for the upregulation of P53 expression in response to DNA damage. It interacts with P53 to enhance its stability and activity by abrogating MDM2 mediated degradation. *NLK* is downregulated in eBL tumor cells and also targeted by 2 miRs that are upregulated in eBL tumor cells. TFAP4/AP4 is a central mediator of cell cycle progression in response to c-MYC activation. **b** RNA seq. Expression counts of *MYC*, *TFAP4*, *ATM* and *NLK* in eBL tumor cells and GC B cells. **c** Hierarchical clustering of eBL and GC B cells based on the expression profiles of *MYC*, *TFAP4*, *ATM* and *NLK* also revealed a clear separation of the two groups. **d**. miRNA seq. Expression counts of hsa-miR-26a-5p, hsa-miR-27b-3p, hsa-miR-30b-5p, miR-17~92-cluster members (hsa-miR-19b-3p, and hsa-miR-92a-3p), and let-7-family miRs (hsa-let-7a-5p, hsa-let-7b-5p, hsa-let-7d-5p, hsa-let-7e-5p, and hsa-let-7 g-5p) in eBL tumor cells and GC B cells

We next considered miRNA-mRNA pairs to be of potential biological significance if the change in the miRNA expression produces a change in mRNA expression in the opposite direction and the magnitude of change is higher than that by chance. We first identified genes targeted by the DE miRNAs in eBL using miRWalk2.0, and of these target genes, we identified the DE validated targets of the aberrantly expressed miRNAs (Additional file 8). 220 miRNA-mRNA pairs were identified. To test if the observed miRNA-mRNA pairs were significant and not due to chance, we performed a permutation test repeated 10,000 times. 181 miRNA-mRNA pairs were then identified, to be of potential biological significance (*p*-value < 0.05) (Additional file 8). Fig. 5 illustrates potential miRNA-mRNA pairs that would influence ATM and NLK function in response to DNA damage to facilitate eBL lymphomagenesis.

**Fig. 6:**
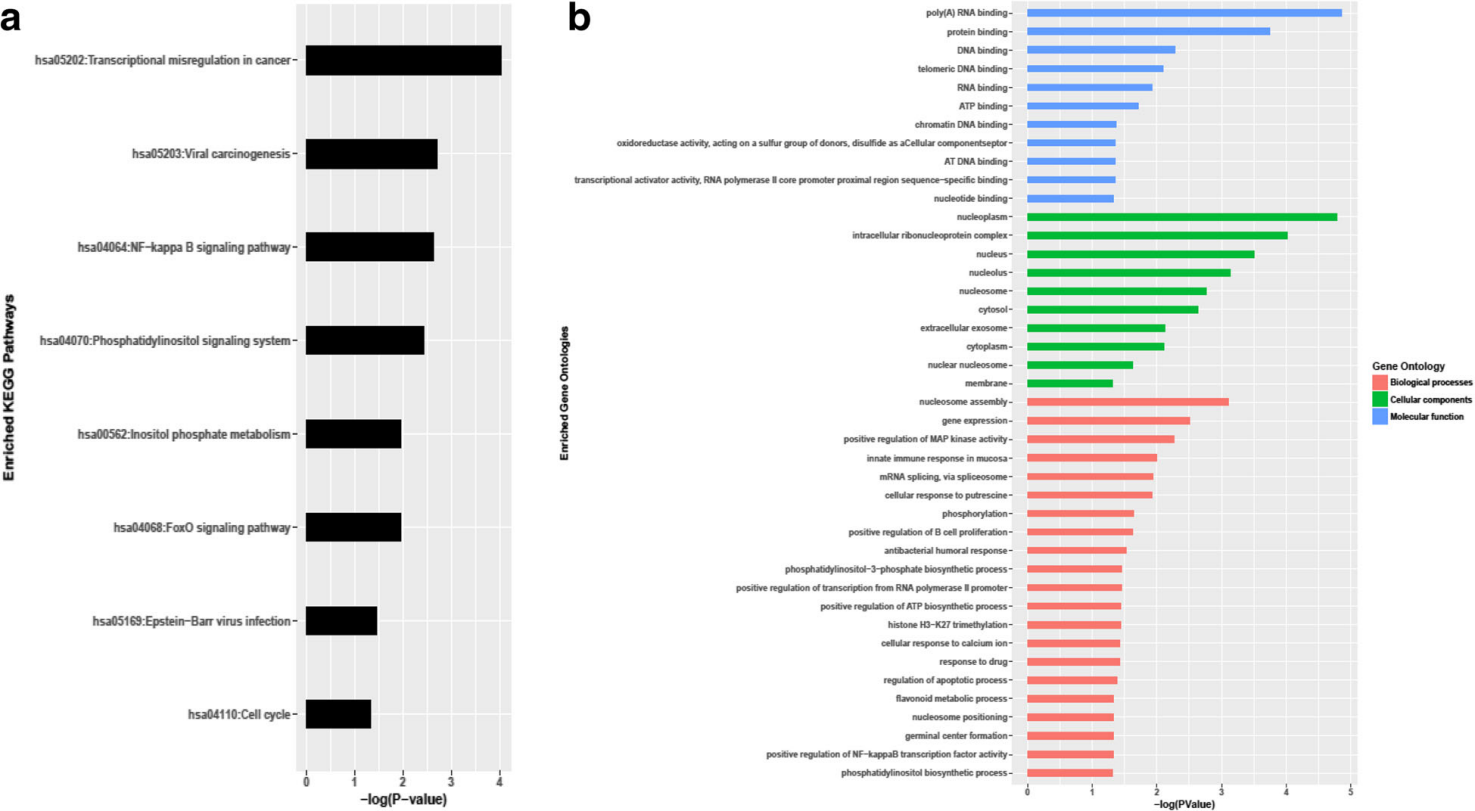
**a** The significantly enriched signaling pathways of the validated target genes of the DE miRNAs that showed an inverse expression change. **b** The significantly enriched gene ontologies (GO’s) of the validated target genes of the DE miRNAs that showed an inverse expression change

Functional enrichment analysis of the inversely-expressed target genes of the DE miRNAs provided us with an overall clue of their functional roles in eBL development. The KEGG pathway analysis demonstrated that the DE targets were significantly associated with transcriptional misregulation, NF-Kappa B signaling, EBV infection, phosphotidlyinisitol signaling and viral carcinogenesis pathways (Fig. 6).

## Discussion

MicroRNAs regulate the expression of approximately 30% of all genes in the human genome [65]. In a normal cell, the interaction of miRNAs and target mRNAs is tightly regulated, whereas this regulation is often lost in cancer cells. A growing body of evidence suggests that miRNAs are aberrantly expressed in many human cancers and that they play significant roles in the initiation and development of these cancers [66]. Therefore, to better understand the specific molecular characteristics of eBL, we identified differentially expressed miRNAs and mRNAs in eBL tumor cells compared to GC B cells based on high-throughput sequencing datasets. This is the first attempt to simultaneously analyze mRNA and miRNA expression profiles in eBL tumor cells compared to their normal counterpart. We identified 211 mRNAs and 49 miRNAs DE with fold changes >2 and *P*-values < 0.01 in eBL tumor cells compared to GC B cells. Of these, 181 miRNA-mRNA pairs, which appeared to be of genuine biological significance and not by chance, showed an inverse direction of expression change. With the aim of understanding the transcriptome expression changes pivotal to eBL development, we identified the aberrant expression of genes (such as *ATM* and *NLK*) and miRNAs (such as let-7 family members and miR-17~92 cluster members) that could endorse eBL lymphoma development and sustain survival of tumor cells in the presence of myc translocation.

Members of the MiR-17~92 cluster gene are the first miRNAs to be implicated in cancer development [67, 68]. This miRNA gene cluster encodes for six distinct miRNAs (miR-17, miR-18a, miR-19a, miR-19b, miR-20a and miR-92) that share the same seed sequence [68]. These miRNAs are frequently over-expressed in other cancers (including multiple B and T cell lymphoid malignancies as well as colorectal cancer, breast cancer, pancreatic cancer, ovarian cancer, lung cancer, and hepatocellular carcinoma) [67, 69] and in BL compared with other non-Hodgkin lymphomas (NHLs) [28, 35, 68, 70]. *MYC* overexpression, because of its translocation to the immunoglobulin locus in BL, enhances the expression of miR-17~92 cluster miRNAs by binding directly to its genomic locus [22, 71] to accelerate carcinogenesis. MiR-17~92 overexpression has been observed previously in sBL tumors [16]. This is consistent with levels in our eBL study. By observing elevated expression of MYC, miR-19b-3p, miR-92a-3p and miR-92b-3p in eBL tumor cells compared to GC B-cells, we confirm that elevated expression of the miR-17~92 cluster miRNAs is a critical feature facilitating eBL lymphomagenesis. Human let-7 family members were also observed to be abnormally expressed in eBL. These related miRNAs act as tumor suppressors, regulators of differentiation and apoptosis, and have been observed to be downregulated in most cancers [72]. Let-7 regulates many transcription factors and oncogenes that play important roles in cell cycle regulation, cell proliferation and apoptosis. These miRNAs have been shown to repress *MYC* [29] controlling proliferation and tumor development. We observed seven let-7 family members (let-7a, let-7b, let-7c, let-7d, let-7e, let-7f, and let-7 g) to be downregulated in eBL tumor cells compared to GC B-cells consistent with their functional role in the genesis and maintenance [29] of eBL tumor cells in the presence of *MYC* deregulation.

Constitutive MYC activity is necessary for all forms of BL [22, 73], however, overexpression of this proto-oncogene also induces apoptotic stress responses which are overcome during lymphomagenesis. Following *MYC* translocation and deregulation in eBL, apart from genetic alterations and mutations that would facilitate escape from myc-mediated apoptosis [73–75], aberrantly expressed miRNAs may also enable a cell to tolerate such oncogene-induced apoptotic stress. MYC is known to activate the p53 tumor suppressor pathway to initiate the apoptotic stress response, however tumor cell survival prevails. The observed downregulation of *ATM* gene and *NLK* (Nemo-like Kinase) in eBL, possibly due to small-interfering RNA mediated regulation, would impair P53 induced by MYC, initiating lymphoma occurrence.

Loss of ATM has been observed in gastric cancer [76, 77]. This checkpoint kinase, transduces genomic stress signals to halt cell cycle progression in response to DNA damage. It is critical in the regulation of the P53 apoptotic pathway and lymphomagenesis in *c-myc* induced lymphomas [78, 79]. ATM could be a pivotal tumor suppressor in response to the translocation occurrence characteristic of eBL tumor cells. It is possible that during tumorigenesis a number of GC B cells have low *ATM* levels due to small interfering RNA-mediated regulation, as a result of irregular expression of miR-27b-3p, miR-26a-5p, miR-30b-5p and myc-dependent activation of miR-17~92 cluster miRNAs. In turn the levels fall below the threshold to halt cell cycle progression in response to DNA damage and maintain P53 activation. Downregulation of *ATM* gene in eBL tumor cells, implies a defective response to DNA damage and P53 activation to suppress tumor development initiated by the *t(8:14)* chromosomal translocation. Upregulation of miRNAs (miR-27b-3p, miR-26a-5p, miR-30b-5p, miR-19b-3p, and miR-92b-3p) in eBL targeting *ATM* suggests abnormal miRNA mediate regulation of this gene which would lead to ATM loss. The observed *NLK* downregulation in eBL tumor cells could also be critical to aid in tumor cell escape from certain death initiated by DNA damage (that results in the *c-myc-Igh* chromosomal translocation) and oncogene-induced apoptotic stress. NLK has been shown to be an important P53 regulator in response to DNA damage and is critical to P53 stability and function [80, 81]. Based on our results, we hypothesize that low *NLK* levels in eBL tumors, probably due to miRNA (upregulated miR-92a-3p and miR-27b-3p expression) mediated regulation, would reduce the stability and activation of P53 in suppressing eBL lymphomagenesis. *ATM* and *NLK* genes were also observed to be significantly down-regulated in established BL cell lines (Namalwa, Raji Ramos, Daudi, Thomas, BL41, BL2, BL30, BL70, CA46, and Gumbus) compared to GC B cells (Additional file 9), supporting the notion that loss of these genes are critical to eBL lymphomagenesis and tumor cell survival.

Our data also revealed the upregulation of *TFAP4/AP4* (transcription factor AP-4) in eBL tumor cells. Interestingly, *AP4* is a c-MYC inducible transcription factor that has been shown to be elevated in many types of tumors [79, 82–85] and it has been shown to also harbor an oncogenic potential [86]. Therefore, it is likely that the upregulation of AP4 expression also mediates cell cycle progression, probably in response to MYC activation, coupled with P53 loss of function due to miRNA regulation of *ATM* and *NLK*, would facilitate the survival of cells harboring the *c-myc-Igh* translocation initiating eBL tumor development (Fig. 5).

EBV is highly associated with eBL diagnosed in Africa and thus the observed enrichment of infection and viral carcinogenesis pathways was not unexpected. Presence of EBV encoded proteins such as EBNA-1, EBNA-3C and LMP-1 promote genomic instability [87], which could contribute to eBL pathogenesis. Genomic instability, which would be initiated by EBV latent proteins coupled with loss of *ATM* as observed and impaired P53 activity (as a result of the observed *NLK* loss) due to miRNA repression, would favor the proliferation and survival of eBL tumor cells. EBV miRNA (ebv-miR-bart5), which is expressed in eBL tumor cells [56], and LMP-1 gene can also inhibit *ATM* expression [87]. However the observed down-regulation of *ATM* in EBV negative BL cell lines (BL2, BL30, BL41, BL70, CA46, Gumbus, and Ramos) (Additional file 9) supports the notion that, irrespective of EBV’s association with eBL, other genetic aberrations could lead to *ATM* loss in eBL. Genomic aberrations such as abnormal upregulation of host miRNAs (miR-27b-3p, miR-26a-5p, miR-30b-5p, miR-19b-3p, and miR-92b-3p) targeting *ATM* would favor proliferation, tumor cell survival and occurrences of mutations that would favor oncogenesis.

## Conclusion

In summary, this study represents the first integrative analysis of miRNA and mRNA expression in eBL tumors. We identified a number of mRNAs and miRNAs that are DE in eBL compared to GC B-cells, the postulated progenitor cell type. The differentially regulated miRNAs and mRNAs identified in eBL contribute to our understanding of the multifactorial nature of eBL lymphomagenesis. We speculate that the combined loss of p53 function in response to DNA damage and oncogene (MYC) induced stress may be due to miRNA-mediated regulation of *ATM* and *NLK* together with upregulation of *TFAP4*. Combined, this facilitates survival of eBL tumor cells with the *c-myc-Igh* chromosomal translocation and promotes MYC induced cell cycle progression initiating eBL lymphomagenesis.

## Additional files

Additional file 1: Principal Component analysis (PCA) using mRNA expression profile. A) PCA plot showing clustering of GC B cells and eBL tumor cells before batch effect removal. B) PCA plot showing clustering of GC B cells and eBL tumor cells after batch effect/noise removal using svaseq. We now observe better clustering of GC B cells based on cell type and not clustering based the previous studies we obtained the data from. (PDF 126 kb)

Additional file 2: Expression of B-cell differentiation markers and eBL diagnostic surface markers. A) Expression of eBL diagnostic surface markers (CD79, CD10, CD20, and CD19). B) Expression of key transcription factors involved in B-cell differentiation (BLIMP1, IRF4, BCL6 and PAX5). (PDF 106 kb)

Additional file 3: Differentially expressed Genes between eBL tumor cells and GC B cells (logFC > 2, *p*-value < 0.01 and FDR < 0.01). (PDF 341 kb)

Additional file 4: Enriched gene sets. (XLSX 11 kb)

Additional file 5: Validated target gene of the DE miRNAs in eBL compared to germinal center B cells. (XLSX 833 kb)

Additional file 6: Enriched gene ontologies and KEGG pathways. (XLSX 208 kb)

Additional file 7: MiRNAs significantly enriched by the network propagation-based method (network perturbation effect score (NPES) >2, adjusted *p*-value < 0.05 and FDR < 0.1) in regulation of the aberrant gene expression profile in eBL. (PDF 25 kb)

Additional file 8: miRNA-mRNA pairs permutation test results. (PDF 302 kb)

Additional file 9: ATM and NLK downregulation in BL cell lines. A.) Hierarchical clustering of BL cell lines and germinal center (GC) B cells based on the expression of MYC, NLK and ATM genes. B.) Expression changes of ATM and NLK in BL cell line compared to GC B cells. (PDF 403 kb)

## Abbreviations

ATM: Ataxia-telangiectasia mutated
BH: Benjamini and Hochberg
BL: Burkitt’s lymphoma
DAVID: Database for Annotation, Visualization and Integrated Discovery
DE: Differentially expressed
eBL: Endemic BL
EBV: Epstein-Barr virus
FC: Fold change
FDR: False discovery rate
GC: Germinal center
KEGG: Kyoto Encyclopedia of Genes and Genomes
miRNA: microRNA
mRNA: messenger RNA
NLK: Nemo-like Kinase
NPES: Network perturbation effect score
