# Supplementary material for: Integrative microRNA and mRNA deep-sequencing expression profiling in endemic Burkitt lymphoma
Source: BMC Cancer. 2017 Nov 13;17:761. doi: 10.1186/s12885-017-3711-9 (PMC5683570; doi:10.1186/s12885-017-3711-9)
Supplement: Supplementary file 1 — Principal Component analysis (PCA) using mRNA expression profile. A) PCA plot showing clustering of GC B cells and eBL tumor cells before batch effect removal. B) PCA plot showing clustering of GC B cells and eBL tumor cells after batch effect/noise removal using svaseq. We now observe better clustering of GC B cells based on cell type and not clustering based the previous studies we obtained the data from. (PDF 126 kb) [file 12885_2017_3711_MOESM1_ESM.pdf]

Additional file 1

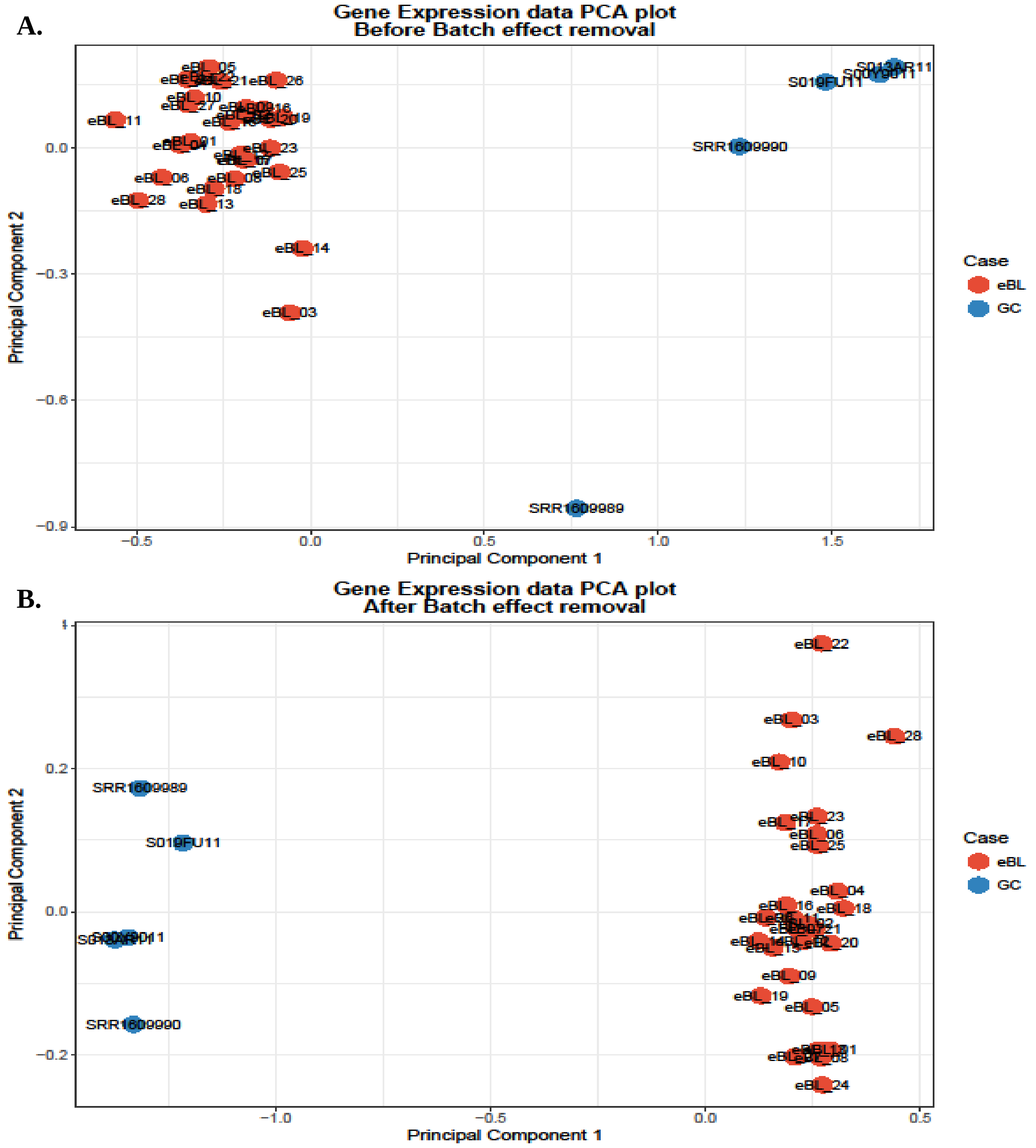

A) PCA plot showing clustering of GC B cells and eBL tumor cells before batch effect removal. **B**) PCA plot showing clustering of GC B cells and eBL tumor cells after batch effect/noise removal using svaseq. We now observe better clustering of GC B cells based on cell type and not clustering based the previous studies we obtained the data from.
