# Supplementary material for: Integrative microRNA and mRNA deep-sequencing expression profiling in endemic Burkitt lymphoma
Source: BMC Cancer. 2017 Nov 13;17:761. doi: 10.1186/s12885-017-3711-9 (PMC5683570; doi:10.1186/s12885-017-3711-9)

# Additional file 2

**A**) Expression of eBL diagnostic surface markers.

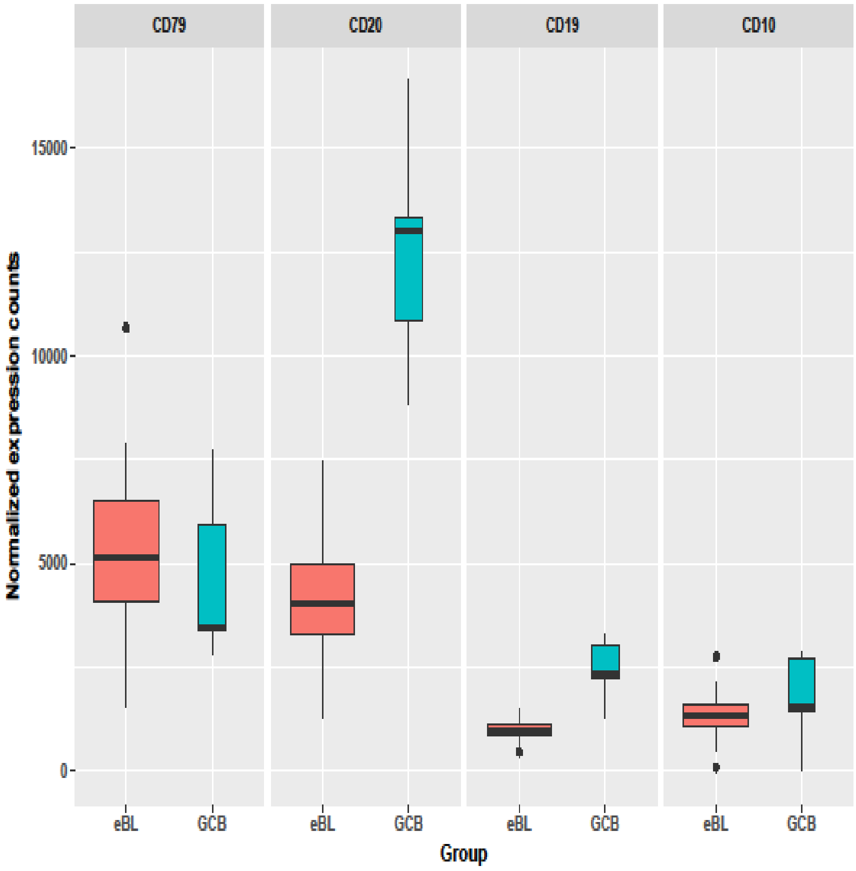


**B**) Expression of key transcription factors involved in B-cell differentiation.

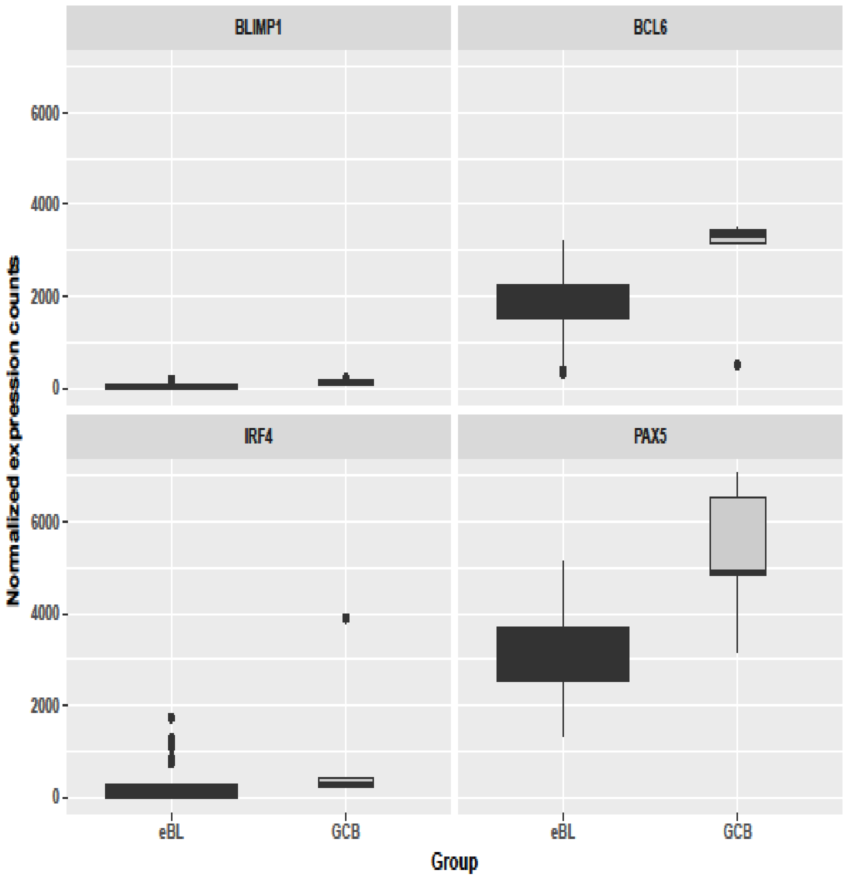

Supplement: Supplementary file 2 — Expression of B-cell differentiation markers and eBL diagnostic surface markers. A) Expression of eBL diagnostic surface markers (CD79, CD10, CD20, and CD19). B) Expression of key transcription factors involved in B-cell differentiation (BLIMP1, IRF4, BCL6 and PAX5). (PDF 106 kb) [file 12885_2017_3711_MOESM2_ESM.pdf]
