# Supplementary material for: Integrative microRNA and mRNA deep-sequencing expression profiling in endemic Burkitt lymphoma
Source: BMC Cancer. 2017 Nov 13;17:761. doi: 10.1186/s12885-017-3711-9 (PMC5683570; doi:10.1186/s12885-017-3711-9)
Supplement: Supplementary file 3 — Differentially expressed Genes between eBL tumor cells and GC B cells (logFC > 2, p-value < 0.01 and FDR < 0.01). (PDF 341 kb) [file 12885_2017_3711_MOESM3_ESM.pdf]

Additional file 3: Differentially expressed Genes between eBL tumor cells and GC B cells (logFC>2, *p*-value<0.01 and FDR<0.01).

Gene symbol	Log FC eBL versus GC B cells	BH adjusted *p*-value	FDR
Upregulated genes			
MT-ND2	5.9357	4.60E-44	4.76E-42
MT-ND1	5.8160	1.43E-56	3.78E-54
MT-ATP6	5.4025	1.68E-30	6.24E-29
MT-ND4	5.2228	2.01E-33	9.30E-32
MT-CYB	4.9183	3.80E-34	1.93E-32
MT-ND3	4.4391	1.23E-42	1.15E-40
MT-ND5	4.3117	9.79E-41	7.60E-39
MT-ND4L	4.2542	4.32E-25	1.03E-23
MT-CO2	4.2428	1.71E-32	7.41E-31
SRM	3.5823	1.65E-40	1.23E-38
FUS	3.3145	4.09E-59	1.33E-56
HK2	3.2878	6.02E-27	1.65E-25
NT5DC2	3.1931	1.06E-36	6.01E-35
ATIC	3.1325	1.21E-42	1.15E-40
ANP32B	3.1142	2.94E-65	1.40E-62
MYC	3.0653	5.50E-24	1.15E-22
RGCC	2.9451	2.69E-26	6.93E-25
FKBP4	2.9411	1.86E-36	1.03E-34
FBL	2.9190	1.51E-62	5.67E-60
HNRNPH3	2.8531	2.67E-54	6.36E-52
FTH1	2.8214	1.32E-19	1.87E-18
MT-CO3	2.8068	6.79E-15	6.00E-14
U2AF1	2.7932	2.18E-63	8.65E-61
MIF	2.7535	6.98E-32	2.90E-30
PPDPF	2.7214	5.45E-14	4.32E-13
PABPC1	2.7203	1.72E-43	1.70E-41
MT-CO1	2.7027	1.13E-16	1.19E-15
ACOT7	2.6869	1.99E-23	3.91E-22
LYAR	2.6680	8.60E-36	4.65E-34
CCDC124	2.5947	1.70E-37	1.03E-35
PFDN2	2.5663	6.84E-23	1.30E-21

Additional file 3 (*Continued*)

**Official gene symbol**	**Log FC eBL versus GC B cells**	**BH adjusted *p*-value**	**FDR**
NPRL3	2.5300	4.83E-33	2.16E-31
HMBS	2.5000	4.87E-39	3.16E-37
GNG7	2.4959	8.30E-15	7.18E-14
TOP1MT	2.4562	1.49E-11	8.87E-11
SOX12	2.4545	3.12E-19	4.29E-18
EIF5B	2.4539	2.83E-38	1.76E-36
TOP1	2.4178	3.53E-43	3.40E-41
CTSD	2.4113	3.08E-08	1.23E-07
CCDC86	2.3837	1.19E-35	6.37E-34
RAB11B	2.3754	6.45E-31	2.45E-29
TXN	2.3506	2.95E-13	2.11E-12
GAR1	2.3093	1.23E-35	6.49E-34
PCBP1	2.2807	5.41E-44	5.52E-42
CD68	2.2736	1.53E-10	8.08E-10
C20orf27	2.2525	2.61E-25	6.26E-24
FTL	2.2334	7.24E-15	6.33E-14
ATP6V0C	2.2316	3.53E-24	7.56E-23
HNRNPA3	2.2102	2.00E-40	1.47E-38
LYZ	2.2069	1.03E-07	3.84E-07
HNRNPD	2.2003	2.40E-38	1.50E-36
UCK2	2.1937	3.11E-10	1.60E-09
NOB1	2.1929	1.81E-36	1.01E-34
NCL	2.1928	3.04E-29	1.01E-27
UTP4	2.1917	1.58E-39	1.07E-37
BANF1	2.1898	1.48E-33	6.99E-32
BMP7	2.1813	9.40E-08	3.52E-07
CMSS1	2.1591	5.69E-17	6.20E-16
PCBP2	2.1560	1.08E-41	9.11E-40
SF3A2	2.1431	1.25E-26	3.29E-25
SLC25A6	2.1372	2.33E-14	1.93E-13
TFAP4	2.1232	1.18E-35	6.35E-34
COPS6	2.0979	5.94E-36	3.24E-34
FAM216A	2.0787	2.56E-29	8.64E-28

Additional file 3 (*Continued*)

Official gene symbol	Log FC eBL versus GC B cells	BH adjusted *p*-value	FDR
CHERP	2.0697	4.87E-42	4.24E-40
LDHA	2.0676	3.68E-19	5.00E-18
TTLL12	2.0642	8.86E-25	2.04E-23
HADH	2.0619	1.52E-29	5.21E-28
NPM3	2.0613	2.31E-15	2.12E-14
FKBP5	2.0583	3.28E-16	3.29E-15
PRKAR1B	2.0560	1.05E-23	2.13E-22
RAB7A	2.0516	1.67E-41	1.34E-39
SRP68	2.0501	5.96E-34	2.96E-32
CYB5A	2.0486	1.93E-09	9.03E-09
TRAP1	2.0446	2.81E-20	4.24E-19
TFDP2	2.0443	9.52E-08	3.56E-07
NME1	2.0346	2.83E-31	1.10E-29
GPATCH4	2.0234	5.09E-18	6.15E-17
NDUFS6	2.0175	4.42E-32	1.88E-30
Downregulated genes			
HIST1H1C	-5.2047	1.08E-102	3.86E-99
ZNF277	-4.9458	5.31E-131	3.79E-127
HIST1H2BC	-4.7110	2.20E-44	2.35E-42
HIST1H1E	-4.5599	7.48E-56	1.84E-53
HIST1H2BD	-4.3218	1.12E-56	3.07E-54
CD83	-3.7611	9.66E-57	2.76E-54
LPP	-3.7011	4.12E-28	1.25E-26
TBC1D8B	-3.4523	1.24E-41	1.01E-39
SULT1B1	-3.3667	7.31E-53	1.49E-50
ASTN2	-3.3030	9.53E-48	1.26E-45
KIAA1328	-3.2938	1.57E-50	2.80E-48
ITPR1	-3.2590	1.64E-48	2.45E-46
HIST1H2BK	-3.2317	1.28E-35	6.72E-34
CD40	-3.2115	1.01E-82	1.45E-79
MORC4	-3.1918	5.71E-44	5.74E-42

Additional file 3 (*Continued*)

Official gene symbol	Log FC eBL versus GC B cells	BH adjusted *p*-value	FDR
SHISA9	-3.1803	4.54E-48	6.49E-46
WDPCP	-3.1656	3.46E-54	7.96E-52
CTSH	-3.0781	5.34E-33	2.37E-31
TRAF1	-3.0247	7.46E-30	2.64E-28
PPP1R15A	-2.9682	2.52E-24	5.51E-23
C4orf32	-2.9633	1.21E-64	5.38E-62
EML6	-2.9589	1.69E-18	2.16E-17
FMNL3	-2.9514	6.22E-34	3.06E-32
PARP15	-2.9012	8.66E-40	6.06E-38
ATP9B	-2.8848	6.99E-81	6.24E-78
ATP8A1	-2.8567	3.86E-49	6.27E-47
SIAH2	-2.8279	1.29E-26	3.39E-25
RPAP2	-2.8176	1.64E-42	1.50E-40
MPV17L	-2.8055	3.65E-33	1.68E-31
DUSP2	-2.7968	9.65E-68	4.92E-65
SLC15A2	-2.7075	1.12E-36	6.29E-35
UGCG	-2.6922	6.17E-50	1.07E-47
FCRL3	-2.6601	3.85E-10	1.95E-09
CNR2	-2.6413	2.43E-24	5.33E-23
SOD2	-2.6365	6.66E-38	4.07E-36
GCNT2	-2.6352	3.23E-64	1.36E-61
LAT2	-2.6183	1.43E-21	2.38E-20
CIITA	-2.6057	2.99E-82	3.56E-79
DMXL1	-2.6054	8.55E-71	5.09E-68
NFAT5	-2.5836	2.10E-47	2.72E-45
KLHL6	-2.5731	2.37E-28	7.43E-27
BMF	-2.5619	1.86E-16	1.91E-15
WDR74	-2.5548	2.07E-37	1.23E-35
SLC9A7	-2.5497	2.25E-44	2.37E-42
NLK	-2.5494	3.82E-22	6.84E-21
MFSD4B	-2.5484	1.95E-44	2.11E-42

Additional file 3 (*Continued*)

Official gene symbol	Log FC eBL versus GC B cells	BH adjusted *p*-value	FDR
LNPEP	-2.5450	3.01E-72	1.95E-69
TSPAN33	-2.5340	7.90E-28	2.34E-26
MEF2C	-2.5067	1.05E-26	2.80E-25
SFT2D2	-2.4950	3.03E-47	3.86E-45
TEP1	-2.4845	1.24E-41	1.01E-39
ATM	-2.4584	7.75E-49	1.18E-46
HLA-DMB	-2.4387	2.48E-21	4.05E-20
LPIN1	-2.4353	2.33E-21	3.81E-20
OTULIN	-2.4171	1.71E-81	1.74E-78
PAG1	-2.4169	5.56E-22	9.77E-21
TMC6	-2.3908	7.61E-34	3.72E-32
ZBTB37	-2.3905	2.24E-53	4.84E-51
MAN2A1	-2.3811	3.03E-30	1.12E-28
BRWD1	-2.3714	8.52E-24	1.75E-22
MACF1	-2.3624	2.23E-23	4.36E-22
ASB3	-2.3477	2.50E-78	1.98E-75
MCTP2	-2.3322	7.89E-28	2.34E-26
OPHN1	-2.3314	1.58E-18	2.03E-17
SETX	-2.3267	1.79E-61	6.07E-59
OXNAD1	-2.3221	1.95E-69	1.07E-66
REL	-2.3205	1.46E-42	1.35E-40
BLOC1S6	-2.3163	3.19E-51	5.83E-49
WDFY4	-2.3045	2.02E-45	2.32E-43
C11orf72	-2.3037	6.66E-26	1.67E-24
VPS13C	-2.2895	7.70E-47	9.48E-45
NEDD9	-2.2881	5.20E-31	1.99E-29
ATAD2B	-2.2862	9.03E-30	3.16E-28
BIRC6	-2.2836	3.20E-97	7.62E-94
RAB8B	-2.2811	2.24E-30	8.28E-29
RBM15	-2.2722	7.50E-78	5.36E-75
SNX11	-2.2719	3.24E-56	8.25E-54
ZNF407	-2.2704	6.55E-88	1.17E-84

Additional file 3 (*Continued*)

Official gene symbol	Log FC eBL versus GC B cells	BH adjusted *p*-value	FDR
KIAA1109	-2.2655	1.23E-51	2.31E-49
CYTH1	-2.2623	9.70E-29	3.15E-27
PTPN6	-2.2551	1.56E-13	1.16E-12
JUNB	-2.2435	9.31E-30	3.24E-28
SLC23A2	-2.2216	9.73E-22	1.65E-20
ANKRD44	-2.2157	5.74E-48	7.73E-46
TMED8	-2.2117	8.92E-28	2.62E-26
STAT3	-2.2113	1.17E-22	2.17E-21
BMP2K	-2.2087	3.70E-48	5.39E-46
ZBTB43	-2.1998	7.75E-42	6.66E-40
VPS13B	-2.1947	6.06E-59	1.88E-56
ZNF141	-2.1742	2.10E-16	2.15E-15
DNAJC10	-2.1648	2.94E-24	6.39E-23
NFKBIE	-2.1622	6.95E-41	5.51E-39
CFLAR	-2.1613	6.25E-32	2.62E-30
SLC35F5	-2.1586	2.77E-29	9.28E-28
IL12RB1	-2.1550	1.31E-23	2.63E-22
SORL1	-2.1449	4.64E-12	2.91E-11
TLE4	-2.1448	4.26E-25	1.02E-23
LCOR	-2.1414	1.18E-45	1.39E-43
CMTM6	-2.1398	2.09E-53	4.67E-51
SMG1	-2.1383	5.90E-62	2.11E-59
HERC1	-2.1363	1.04E-39	7.04E-38
SAMD8	-2.1061	2.44E-45	2.77E-43
ITGAL	-2.0953	4.84E-21	7.67E-20
RAB11FIP1	-2.0934	1.25E-15	1.17E-14
MYO1E	-2.0826	5.37E-09	2.37E-08
PIK3CG	-2.0811	2.55E-27	7.24E-26
ITPKB	-2.0741	7.00E-18	8.38E-17
MALT1	-2.0723	1.32E-29	4.52E-28
TACC1	-2.0676	5.67E-25	1.32E-23
ZNF780A	-2.0604	3.77E-57	1.12E-54

Additional file 3 (*Continued*)

**Official gene symbol**	**Log FC eBL versus GC B cells**	**BH adjusted *p*-value**	**FDR**
LHFPL2	-2.0599	3.46E-11	1.97E-10
TTLL3	-2.0568	1.96E-08	8.05E-08
PIKFYVE	-2.0545	3.54E-46	4.29E-44
DCP2	-2.0539	9.41E-25	2.15E-23
DOPEY1	-2.0539	4.83E-39	3.16E-37
MTMR14	-2.0514	3.00E-43	2.93E-41
PHF6	-2.0513	5.76E-45	6.43E-43
SNX29	-2.0407	2.64E-40	1.90E-38
SLC12A2	-2.0393	1.84E-33	8.60E-32
KATNAL1	-2.0385	1.79E-26	4.69E-25
RELT	-2.0343	3.31E-29	1.09E-27
IRGQ	-2.0287	7.49E-23	1.41E-21
RNF19A	-2.0278	4.32E-40	3.09E-38
INO80D	-2.0251	1.13E-40	8.59E-39
ORC4	-2.0249	6.13E-30	2.18E-28
PASK	-2.0244	6.28E-23	1.20E-21
CYB561A3	-2.0195	8.56E-27	2.30E-25
LRRC37A2	-2.0179	5.80E-37	3.40E-35
RABGAP1L	-2.0090	1.11E-24	2.51E-23
CDK17	-2.0075	4.62E-33	2.09E-31
PHC3	-2.0040	7.32E-53	1.49E-50
ITSN2	-2.0011	1.09E-21	1.83E-20

Abbreviations: eBL, endemic Burkitt lymphoma; GC, germinal center; BH, Benjamini & Hochberg; FC, Fold Change; FDR, False discovery rate.
