# Supplementary material for: Integrative microRNA and mRNA deep-sequencing expression profiling in endemic Burkitt lymphoma
Source: BMC Cancer. 2017 Nov 13;17:761. doi: 10.1186/s12885-017-3711-9 (PMC5683570; doi:10.1186/s12885-017-3711-9)
Supplement: Supplementary file 7 — MiRNAs significantly enriched by the network propagation-based method (network perturbation effect score (NPES) >2, adjusted p-value < 0.05 and FDR < 0.1) in regulation of the aberrant gene expression profile in eBL. (PDF 25 kb) [file 12885_2017_3711_MOESM7_ESM.pdf]

Additional file 7: MiRNAs significantly enriched by the network propagation-based method (network perturbation effect score (NPES) >2, adjusted *p*-value<0.05 and FDR<0.1) in regulation of the aberrant gene expression profile in eBL

MiRFam	DE miRNAs	NPES.zscore	*p*-value	FDR
miR-19ab	hsa-miR-19b-3p	2.8993	**0.001**	**0.01837**
miR-25/32/92abc/363/363-3p/367	hsa-miR-92a-3p, hsa-miR-92b-3p	3.9415	**0.001**	**0.01837**
miR-29abcd	hsa-miR-29a-3p, hsa-miR-29c-3p	3.0281	**0.002**	**0.02673**
miR-140/140-5p/876-3p/1244	hsa-miR-140-3p	3.1259	**0.003**	**0.0294**
miR-183	hsa-miR-183-5p	2.6688	**0.005**	**0.04594**
miR-26ab/1297/4465	hsa-miR-26a-5p	2.6475	**0.006**	**0.049**
miR-221/222/222ab/1928	hsa-miR-221-3p, hsa-miR-222-3p	2.5557	**0.007**	**0.049**
miR-182	hsa-miR-182-5p	2.6168	**0.008**	**0.05113**
miR-27abc/27a-3p	hsa-miR-27b-3p	2.4344	**0.011**	**0.06468**
let-7/98/4458/4500	let-7a-5p, let-7b-5p, let-7c, let-7d-5p, let-7e-5p, let-7f-5p, let-7g-5p	2.2441	**0.013**	**0.06825**
miR-30abcdef/30abe-5p/384-5p	hsa-miR-30b-5p, hsa-miR-30e-5p, hsa-miR-30e-3p	2.3349	**0.013**	**0.06825**
miR-17/17-5p/20ab/20b-5p/93/106ab/427/518a-3p/519d	hsa-miR-20a-5p	2.1264	**0.019**	**0.0735**

Additional file 7 (*Continued*)

**MiRFam**	**DE miRNAs**	**NPES.zscore**	***p*-value**	**FDR**
miR-22/22-3p	hsa-miR-22-3p	1.8841	0.032	0.1045
miR-148ab-3p/152	hsa-miR-148a-3p	1.8413	0.033	0.1055
miR-186	hsa-miR-186-5p	1.8805	0.034	0.1063
miR-24/24ab/24-3p	hsa-miR-24-3p	1.8591	0.041	0.1159
miR-21/590-5p	hsa-miR-21-3p	1.8408	0.044	0.1198
miR-130ac/301ab/301b/301b-3p/454/721/4295/3666	hsa-miR-130a-3p	1.7793	0.045	0.1203
miR-10abc/10a-5p	hsa-miR-10a-5p	1.5474	0.062	0.1364
miR-486-5p/3107	hsa-miR-486-5p	1.3684	0.102	0.1874
miR-103a/107/107ab	hsa-miR-103a-3p,	1.2289	0.115	0.2037
miR-15abc/16/16abc/195/322/424/497/1907	hsa-miR-16-5p	0.9955	0.165	0.2445
miR-340-5p	hsa-miR-340-5p	0.7337	0.224	0.294
miR-378/422a/378bcdefhi	hsa-miR-378i	0.4574	0.312	0.3729
miR-146ac/146b-5p	hsa-miR-146b-5p	0.2910	0.37	0.4216
miR-320abcd/4429	hsa-miR-320a, hsa-320b	0.2399	0.4	0.4421
miR-28-5p/708/1407/1653/3139	hsa-miR-28-5p	-0.4186	0.651	0.6692

Abbreviations: MiRFam, miRNA Family; DE, differentially expressed; NPES, network perturbation enrichment score; FDR, False discovery rate
