# Supplementary material for: Integrative microRNA and mRNA deep-sequencing expression profiling in endemic Burkitt lymphoma
Source: BMC Cancer. 2017 Nov 13;17:761. doi: 10.1186/s12885-017-3711-9 (PMC5683570; doi:10.1186/s12885-017-3711-9)
Supplement: Supplementary file 8 — miRNA-mRNA pairs permutation test results. (PDF 302 kb) [file 12885_2017_3711_MOESM8_ESM.pdf]

Additional file 8: miRNA-mRNA pairs permutation test results

**DE miRNA**	**Validated DE Targets**	**Permutation test *p*-value**
hsa-miR-16-5p	*CDK17, CYB561A3, DNAJC10, HIST1H1C, HIST1H2BC, HIST1H2BK, INO80D, ITPR1, KATNAL1, MACF1, ORC4, OXNAD1, PAG1, PHC3, REL, RELT, SLC12A2, SNX11, TEP1, TLE4*	**<0.00001**
hsa-miR-92a-3p	***ATM**, CMTM6, DCP2, DMXL1, HERC1, HIST1H1E, IRGQ, ITPR1, KIAA1109, LCOR, LHFPL2, LPIN1, LRRC37A2, MACF1, MAN2A1, MEF2C, MTMR14, RAB8B, REL, SMG1, STAT3, TACC1, ZNF277*	**<0.00001**
hsa-miR-20a-5p	*DNAJC10, DUSP2, FMNL3, HIST1H2BD, ITPKB, KATNAL1, NFAT5, PHF6, RAB11FIP1, RABGAP1L, SAMD8, SLC35F5, SOD2, STAT3, UGCG, VPS13C, ZBTB37, ZNF780A*	**0.0001**
hsa-miR-186-5p	*BLOC1S6, HIST1H1C, HIST1H1E, HIST1H2BK, ITPKB, ITSN2, KATNAL1, PHC3, SFT2D2, TLE4, VPS13B, VPS13C*	**0.0002**
hsa-miR-92b-3p	*ITPR1, KIAA1109, LCOR, LHFPL2, MAN2A1, **NLK**, RAB8B, REL, SMG1, TACC1, ZNF277*	**0.0005**
hsa-miR-19b-3p	***ATM**, BRWD1, BRWD1, DCP2, ITPR1, KATNAL1, MACF1, MALT1, RAB8B, SAMD8, SMG1*	**0.0012**
hsa-miR-320a	*ANP32B, C20orf27, MIF, **MYC**, NPM3, PCBP2, SRM, SRP68*	**0.0042**
hsa-let-7b-5p	*BMP7, GPATCH4, **MYC**, NT5DC2, PABPC1, PCBP2, TTLL12, UCK2*	**0.0045**
hsa-miR-30e-5p	*ASB3, **ATM**, LCOR, LHFPL2, NFAT5, OPHN1, TBC1D8B, VPS13B*	**0.0052**
hsa-miR-30b-5p	*ASB3, **ATM**, BLOC1S6, LCOR, LHFPL2, MYO1E, NFAT5, OPHN1*	**0.0057**

Additional file 8 (*Continued*)

DE miRNA	Validated DE Targets	Permutation test *p*-value
hsa-miR-423-5p	*C20orf27, FKBP4,* ***MYC****, PABPC1, PCBP1, SOX12, SRM*	**0.0077**
hsa-miR-27b-3p	*HIST1H1C, ITSN2, LCOR, LPIN1,* ***NLK****, UGCG*	**0.0155**
hsa-miR-340-5p	*DUSP2, INO80D, ITPR1, KIAA1109, SOD2, SULT1B1*	**0.017**
hsa-miR-10a-5p	*IRGQ, KLHL6, PIK3CG, SFT2D2, TSPAN33, WDR74*	**0.0187**
hsa-miR-24-3p	*BMP7, LDHA,* ***MYC****, TFDP2, TOP1, UCK2*	**0.0196**
hsa-miR-221-3p	*FUS, HNRNPD, NCL, NT5DC2, SRP68*	**0.0342**
hsa-miR-26a-5p	***ATM****, BMP2K, CFLAR, MAN2A1, PIKFYVE*	**0.0342**
hsa-miR-183-5p	*BRWD1, ITPKB, RPAP2, SMG1, TMED8*	**0.0369**
hsa-miR-222-3p	*HNRNPD,* ***MYC****, PCBP2, TRAP1, TXN*	**0.0371**
hsa-let-7e-5p	*MYC, NT5DC2, PCBP2, TTLL12*	0.0726
hsa-miR-1260b	*FUS, HNRNPA3, TFDP2*	0.1296
hsa-miR-130a-3p	*BRWD1, RAB11FIP1, SAMD8*	0.1344
hsa-miR-331-3p	*FUS, LDHA, TRAP1*	0.1352
hsa-miR-182-5p	*BRWD1, FMNL3, MALT1*	0.1369
hsa-miR-320b	*MYC, NPM3, SRP68*	0.142
hsa-miR-21-3p	*C4orf32, DNAJC10*	0.2592
hsa-miR-30e-3p	*CCDC86, MYC*	0.2612
hsa-miR-140-3p	*TTLL12, UCK2*	0.2646

Additional file 8 (*Continued*)

DE miRNA	Validated DE Targets	Permutation test *p*-value
hsa-let-7f-5p	*COPS6*, *MYC*	0.2677
hsa-miR-532-5p	*MAN2A1*	0.5073
hsa-miR-148a-3p	*LNPEP*	0.5084
hsa-miR-660-5p	*UGCG*	0.51
hsa-miR-1260a	*TFDP2*	0.5144
hsa-let-7a-5p	*MYC*	0.5159
hsa-miR-103a-3p	*C20orf27*	0.5192
hsa-let-7g-5p	*MYC*	0.5202
hsa-miR-486-5p	*CD40*	0.5247
hsa-miR-769-5p	*TRAF1*	0.5248
hsa-let-7d-5p	*MYC*	0.5276

Abbreviation: DE, differentially expressed.
