# Supplementary material for: Integrative microRNA and mRNA deep-sequencing expression profiling in endemic Burkitt lymphoma
Source: BMC Cancer. 2017 Nov 13;17:761. doi: 10.1186/s12885-017-3711-9 (PMC5683570; doi:10.1186/s12885-017-3711-9)

Additional file 9

A.) Hierarchical clustering of BL cell lines and germinal center (GC) B cells based on the expression of *MYC*, *NLK* and *ATM* genes.

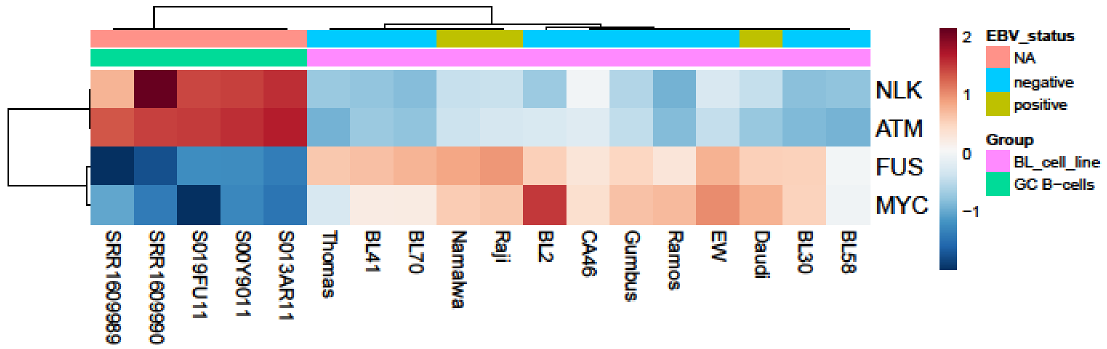

B.) Expression changes of *ATM* and *NLK* in BL cell line compared to GC B cells.

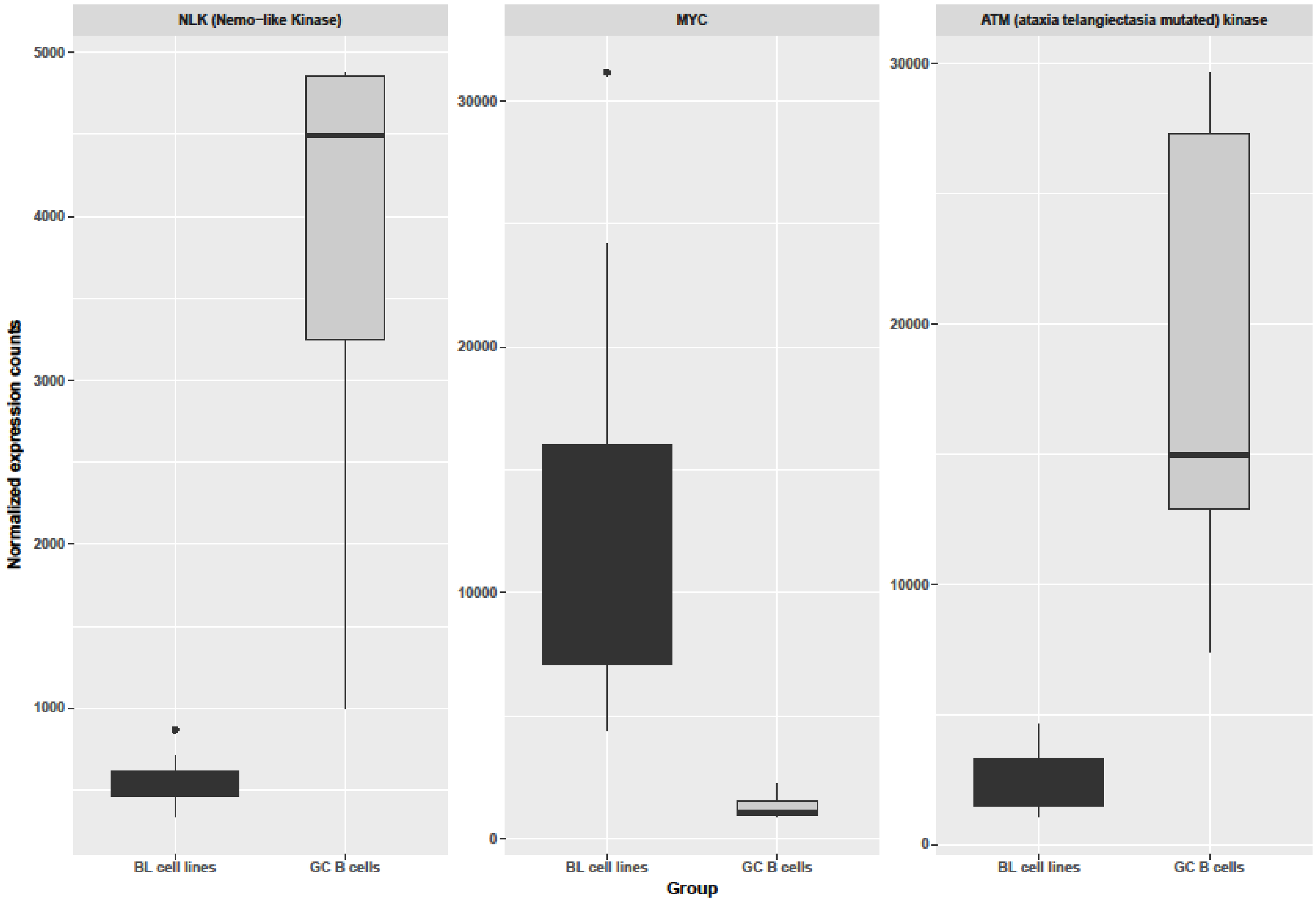

Supplement: Supplementary file 9 — ATM and NLK downregulation in BL cell lines. A.) Hierarchical clustering of BL cell lines and germinal center (GC) B cells based on the expression of MYC, NLK and ATM genes. B.) Expression changes of ATM and NLK in BL cell line compared to GC B cells. (PDF 403 kb) [file 12885_2017_3711_MOESM9_ESM.pdf]
